# The gene expression profiles in response to 102 traditional Chinese medicine (TCM) components: a general template for research on TCMs

**DOI:** 10.1038/s41598-017-00535-8

**Published:** 2017-03-23

**Authors:** Chao Lv, Xueting Wu, Xia Wang, Juan Su, Huawu Zeng, Jing Zhao, Shan Lin, Runhui Liu, Honglin Li, Xuan Li, Weidong Zhang

**Affiliations:** 1 School of Pharmacy, Second Military Medical University, Shanghai, 200433 P.R. China; 2 Shanghai Institute of Pharmaceutical Industry, China State Institute of Pharmaceutical Industry, Shanghai, 201203 P.R. China; 3 Key Laboratory of Synthetic Biology, CAS Center for Excellence in Molecular Plant Sciences, Institute of Plant Physiology and Ecology, Shanghai Institutes for Biological Sciences, Chinese Academy of Sciences, Shanghai, 200032 P.R. China; 4 State Key Laboratory of Bioreactor Engineering, Shanghai Key Laboratory of New Drug Design, School of Pharmacy, East China University of Science and Technology, Shanghai, 200237 P.R. China; 5 Department of Mathematics, Logistical Engineering University, Chongqing, 400016 P.R. China

## Abstract

Traditional Chinese medicines (TCMs) have important therapeutic value in long-term clinical practice. However, because TCMs contain diverse ingredients and have complex effects on the human body, the molecular mechanisms of TCMs are poorly understood. In this work, we determined the gene expression profiles of cells in response to TCM components to investigate TCM activities at the molecular and cellular levels. MCF7 cells were separately treated with 102 different molecules from TCMs, and their gene expression profiles were compared with the Connectivity Map (CMAP). To demonstrate the reliability and utility of our approach, we used nitidine chloride (NC) from the root of *Zanthoxylum nitidum*, a topoisomerase I/II inhibitor and α-adrenoreceptor antagonist, as an example to study the molecular function of TCMs using CMAP data as references. We successfully applied this approach to the four ingredients in Danshen and analyzed the synergistic mechanism of TCM components. The results demonstrate that our newly generated TCM data and related methods are valuable in the analysis and discovery of the molecular actions of TCM components. This is the first work to establish gene expression profiles for the study of TCM components and serves as a template for general TCM research.

## Introduction

Traditional Chinese medicine (TCM), a system of ancient medical practices that differs in methodology and philosophy from modern medicine, plays an important role in health maintenance for the peoples of Asia and is considered a complementary or alternative medical system in most Western countries^1^. Despite the important therapeutic value of TCMs, great challenges remain in understanding the scientific basis of TCMs at the molecular level and from a systemic perspective. The recent application of state-of-the-art technologies in chemical biology to characterize commonly used TCM formulae has provided the means to identify biological targets for the active ingredients in TCMs^2, 3^. However, as TCMs contain a large number of ingredients and many of the active ingredients of TCMs have effects on multiple diseases, the combinatorial rules and roles of most TCM formulae in complex diseases remain to be elucidated.

Recently, there has been growing interest in incorporating expression microarrays as an effective technology for drug development. Since the emergence of the Connectivity Map (CMAP), a novel pathway-independent approach employing gene expression profiles, numerous achievements have been made in the field of drug repurposing, target discovery and elucidating mechanisms of action^4^. The CMAP database is a collection of gene expression profiles from cultured human cell lines treated with drugs. Moreover, pattern-matching software was applied to mine these data and compare gene expression signatures to identify connections among small molecules, genes and diseases^5, 6^. The current version of the CMAP (build 02) contains 6100 expression profiles reflecting 1309 bioactive compounds (http://www.broadinstitute.org/cmap/). To date, the CMAP database has been employed in several studies of TCMs. In a recent study published in *Cell*, Liu *et al*.^7^ employed the CMAP database to identify candidate drugs for the treatment of obesity. Celastrol, a pentacyclic triterpene extracted from the roots of *Tripterygium wilfordii* (thunder god vine) plant, increases leptin sensitivity to suppress food intake and dramatically reduce body weight in obese mice. Wen *et al*. used the CMAP database^8^ to identify the model TCM formula Si-Wu-Tang (SWT), which is widely used for women’s health, as a nuclear factor erythroid 2-related factor 2 (Nrf2) activator and phytoestrogen. These studies demonstrate the feasibility of combining microarray-based gene expression profiles with CMAP mining to elucidate the mechanisms of action and discover the targets/pathways of TCM components.

However, compounds in the CMAP database mainly include US Food and Drug Administration (FDA)-approved and experimental drugs, most of which are not derived from TCMs, and research on the gene expression profiles of TCM molecules has been sparse. Thus, the current work seeks to establish the public and unified gene expression profiles of TCM components constructed according to the CMAP database. Gene expression profiles were produced from a human breast cancer epithelial cell line (MCF7) treated with 102 TCM ingredients to clarify the effects of TCM molecules on gene expression levels. The elucidation of the gene expression profiles, targets/pathways of small molecules, and mechanisms of activity of Chinese herbs and TCM formulae by combining the CMAP database and other bioinformatics methods will contribute to the efficacy of pharmacological prediction and drug discovery. As described below, we have employed gene expression profiles to mine molecular functions of TCM components and elucidate the synergistic mechanisms of TCM molecules, and the analytical results were validated with functional experiments.

## Results

### Generation of gene expression profiles for 102 TCM components

**Figure 1:**
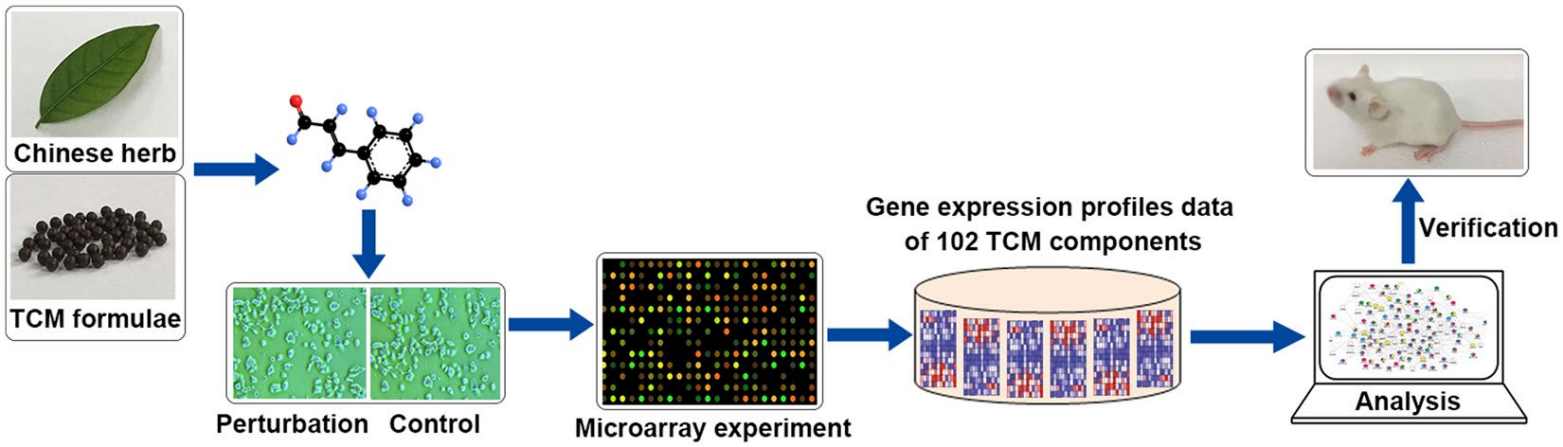
The schematic view of data construction and process.

The schematic view of data construction and processing is presented in Fig. 1. All molecules were derived from TCMs and were mainly active ingredients in Chinese herbs and TCM formulae. MCF7 cells were then treated with the derived molecules and total RNA was extracted for microarray analysis. Finally, the gene expression profiles of TCM components were established for the study of TCMs. It is possible that some gene expression profiles of TCM ingredients have been reported in previous studies. However, we produced the gene expression profiles on a unified platform that was more conducive to the collective analysis of the different ingredients. In total, 102 components are provided in Table 1. The raw data of gene expression profiles of TCM components are available through the National Center for Biotechnology Information’s Gene Expression Omnibus (GEO, http://www.ncbi.nlm.nih.gov/geo/), and the GEO series accession number is GSE85871. The gene expression profile data can be analyzed in combination with public database CMAP and other bioinformatics methods.

**Table 1:** The list of 102 molecules in TCMs.

NO.	Molecules	Molecular formula	Concentration	NO.	Molecules	Molecular formula	Concentration
1	Glycyrrhizic acid	C_42_H_62_O_16_	10 μM	52	1β-hydroxyalantolactone	C_15_H_20_O_3_	10 μM
2	Hydroxysafflor yellow A	C_27_H_32_O_16_	10 μM	53	Salvianic acid A sodium	C_9_H_10_O_5_	10 μM
3	Anhydroicaritin	C_21_H_20_O_6_	10 μM	54	Isoalantolactone	C_15_H_20_O_2_	10 μM
4	Hyperoside	C_21_H_20_O_12_	10 μM	55	Alantolactone	C_15_H_20_O_2_	10 μM
5	Hesperidin	C_28_H_34_O_15_	10 μM	56	Resibufogenin	C_24_H_32_O_4_	1 μM
6	Puerarin	C_21_H_20_O_9_	10 μM	57	Bufalin	C_24_H_34_O_4_	1 μM
7	Aconitine	C_34_H_47_NO_11_	10 μM	58	Arenobufagin	C_24_H_32_O_6_	1 μM
8	Stachydrine hydrochloride	C_7_H_14_ClNO_2_	10 μM	59	Cinobufagin	C_26_H_34_O_6_	1 μM
9	Ephedrine hydrochloride	C_10_H_16_ClNO	10 μM	60	Bufotoxin	C_40_H_60_N_4_O_10_	1 μM
10	Berberine	C_20_H_18_NO_4_^+^	10 μM	61	Telocinobufagin	C_24_H_34_O_5_	1 μM
11	Ginkgolide B	C_20_H_24_O_10_	10 μM	62	Bufotaline	C_26_H_36_O_6_	1 μM
12	Bilobalide	C_15_H_18_O_8_	10 μM	63	Cinobufotalin	C_26_H_34_O_7_	1 μM
13	Andrographolide	C_20_H_30_O_5_	10 μM	64	Salidroside	C_14_H_20_O_7_	10 μM
14	Paeoniflorin	C_23_H_28_O_11_	10 μM	65	Daidzin	C_21_H_20_O_9_	10 μM
15	Tanshinone IIA	C_19_H_18_O_3_	10 μM	66	Schisantherin A	C_30_H_32_O_9_	10 μM
16	Lobetyolin	C_20_H_28_O_8_	10 μM	67	Schizandrin	C_24_H_32_O_7_	10 μM
17	Emodin	C_15_H_10_O_5_	10 μM	68	Oxymatrine	C_15_H_24_N_2_O_2_	10 μM
18	Ginsenoside Rg1	C_42_H_72_O_14_	10 μM	69	Matrine	C_15_H_24_N_2_O	10 μM
19	Ginsenoside Rb1	C_54_H_92_O_23_	10 μM	70	Osthole	C_15_H_16_O_3_	10 μM
20	Astragaloside IV	C_41_H_68_O_14_	10 μM	71	Silybin	C_25_H_22_O_10_	10 μM
21	Saikosaponin A	C_42_H_68_O_13_	10 μM	72	Oleanic acid	C_30_H_48_O_3_	10 μM
22	Saikosaponin D	C_42_H_68_O_13_	10 μM	73	Phillyrin	C_27_H_34_O_11_	10 μM
23	Salvianolic acid B	C_36_H_30_O_16_	10 μM	74	Curculigoside	C_22_H_26_O_11_	10 μM
24	Chlorogenic acid	C_16_H_18_O_9_	10 μM	75	Scutellarein	C_15_H_10_O_6_	10 μM
25	Ferulic acid	C_10_H_10_O_4_	10 μM	76	β-ecdysterone	C_27_H_44_O_7_	10 μM
26	Gastrodin	C_13_H_18_O_7_	10 μM	77	Strychnine	C_21_H_22_N_2_O_2_	10 μM
27	Acteoside	C_29_H_36_O_15_	10 μM	78	Magnolol	C_18_H_18_O_2_	10 μM
28	Imperatorin	C_16_H_14_O_4_	10 μM	79	Honokiol	C_18_H_18_O_2_	10 μM
29	Artemisinin	C_15_H_22_O_5_	10 μM	80	Geniposide	C_17_H_24_O_10_	10 μM
30	Resveratrol	C_14_H_12_O_3_	10 μM	81	Gallic acid	C_7_H_6_O_5_	10 μM
31	Hyodeoxycholic acid	C_24_H_40_O_4_	10 μM	82	Notoginsenoside R1	C_47_H_80_O_18_	10 μM
32	Deoxycholic acid	C_24_H_40_O_4_	10 μM	83	Liquiritin	C_21_H_22_O_9_	10 μM
33	Ursodeoxycholic acid	C_24_H_40_O_4_	10 μM	84	L-scopolamine	C_17_H_21_NO_4_	10 μM
34	Chenodeoxycholic acid	C_24_H_40_O_4_	10 μM	85	Gentiopicroside	C_16_H_20_O_9_	10 μM
35	Cholic acid	C_24_H_40_O_5_	10 μM	86	Benzoylhypaconitine	C_31_H_43_NO_9_	10 μM
36	Cinnamic acid	C_9_H_8_O_2_	10 μM	87	Benzoylaconitine	C_32_H_45_NO_10_	10 μM
37	Cinnamaldehyde	C_9_H_8_O	10 μM	88	Tetrahydropalmatine	C_21_H_25_NO_4_	10 μM
38	Muscone	C_16_H_30_O	10 μM	89	Hypaconitine	C_34_H_45_NO_11_	10 μM
39	Isoborneol	C_10_H_18_O	10 μM	90	Glycyrrhizic acid	C_42_H_62_O_16_	10 μM
40	Borneol	C_10_H_18_O	10 μM	91	6-gingerol	C_17_H_26_O_4_	10 μM
41	Benzyl benzoate	C_14_H_12_O_2_	10 μM	92	Macrozamin	C_13_H_24_N_2_O_11_	10 μM
42	Ginsenoside Rb3	C_53_H_90_O_22_	10 μM	93	Sennoside A	C_42_H_38_O_20_	10 μM
43	Ginsenoside Rc	C_53_H_90_O_22_	10 μM	94	Oridonin	C_20_H_28_O_6_	1 μM
44	Ginsenoside Rb2	C_53_H_90_O_22_	10 μM	95	Dioscin	C_45_H_72_O_16_	1 μM
45	Ginsenoside Re	C_48_H_82_O_18_	10 μM	96	(+) 2-(1-hydroxyl-4-oxocyclohexyl) ethyl caffeate	C_17_H_20_O_6_	10 μM
46	Ginsenoside Rd	C_48_H_82_O_18_	10 μM	97	Bruceine D	C_20_H_26_O_9_	10 μM
47	Nitidine chloride	C_21_H_18_ClNO_4_	10 μM	98	Narciclasine	C_14_H_13_NO_7_	10 μM
48	Protocatechuic aldehyde	C_7_H_6_O_3_	10 μM	99	Santonin	C_15_H_18_O_3_	10 μM
49	Britanin	C_19_H_26_O_7_	10 μM	100	Ainsliadimer A	C_30_H_34_O_7_	1 μM
50	Japonicone A	C_32_H_40_O_7_	10 μM	101	Chelerythrine	C_21_H_18_NO_4_^+^	1 μM
51	Bacopaside I	C_46_H_74_O_20_S	10 μM	102	Sanguinarine	C_20_H_14_NO_4_^+^	1 μM

### Analysis of TCM component activities

**Figure 2:**
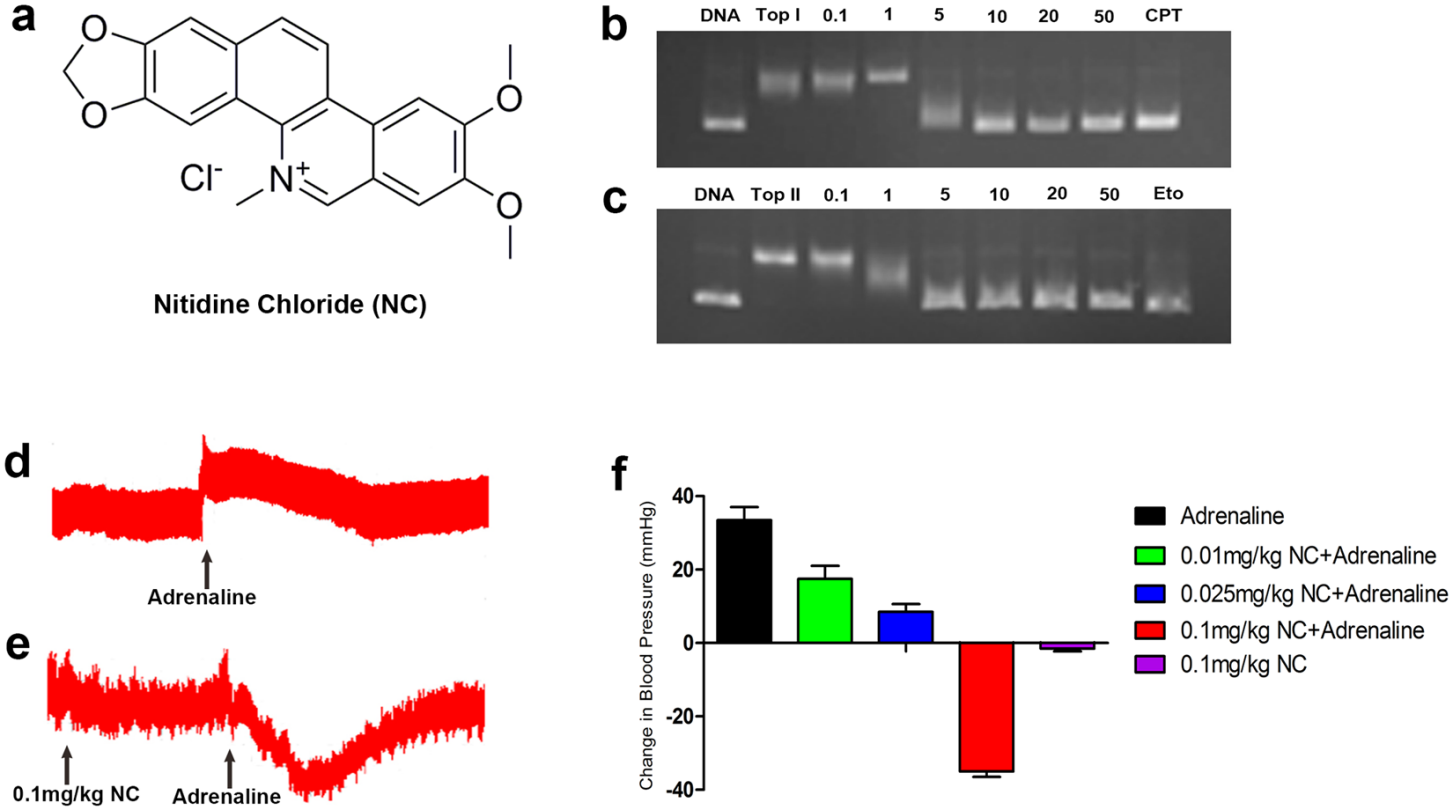
The results of validation experiments. (**a**) Chemical structure of NC. (**b**) Effect of NC on TopI mediated DNA relaxation indifferent concentration. Lane 1, supercoiled pBR322 plasmid DNA; lane 2, DNA + TopI; lane 3–8, DNA + TopI + NC (0.1, 1, 5, 10, 20, 50 μM); lane 9, DNA + TopI + 50 μM camptothecin. (**c**) Inhibition of TopII relaxation activity. Lane 1, supercoiled pBR322 plasmid DNA; lane 2, DNA + TopII; lane 3–8, DNA + TopII + NC (0.1, 1, 5, 10, 20, 50 μM); lane 9, DNA + TopII + 50 μM etoposide. (**d**) The effect of adrenaline (5 μg/kg) on arterial blood pressure. (**e**) NC (0.1 mg/kg) caused reversal of the adrenaline pressor response. (**f**) The effect of NC (0.01, 0.025, 0.1 mg/kg) on the arterial blood pressure response to adrenaline.

We first performed a comparison analysis between the gene expression profiles in response to TCM components and the CMAP database to discover their molecular functions. Nitidine chloride (NC, Fig. 2a) is a natural phytochemical alkaloid and a major active compound isolated from the well-known traditional Chinese medicinal herb *Zanthoxylum nitidum* (Roxb.) DC. Previous studies have reported that NC is a potential anti-tumour drug via the modulation of multiple targets/pathways^9–11^. In the present study, the gene expression profiles of NC-treated MCF7 cells were selected to search the CMAP database. The query signature consisted of 752 genes (173 up-regulated and 579 down-regulated; Supplementary Data 1) that were simultaneously submitted to the CMAP database for analysis.

The similarity between the gene expression profiles of the query signature and a CMAP instance was measured using the connectivity score (from −1 to 1). A highly positive connectivity score indicates inducement of the expression of the query signature by the corresponding drug. The CMAP yielded highly positive connectivity scores for NC-treated MCF7 cells. In the detailed results of CMAP analysis, the top 10 instances of positive correlations are presented in Table 2. The results revealed a total of five compounds in the top ten instances, including irinotecan, phenoxybenzamine, hycanthone, camptothecin, and daunorubicin. Among these five compounds, irinotecan and camptothecin are topoisomerase I inhibitors, and daunorubicin is a topoisomerase II inhibitor, as reported in previous studies^12–14^. Furthermore, phenoxybenzamine is a known α-adrenoreceptor antagonist in the treatment of hypertension, but there have been no reports that NC acts as an α-adrenoreceptor antagonist. An extension of this finding would be to hypothesize that NC might perform activities based on same or similar activities as the compounds with the highest positive connectivity scores.

**Table 2:** Top CMAP analysis of nitidine chloride in detailed results.

Rank	Batch	Cmap name	Dose	Cell	Score
1	1090	irinotecan	100 µM	MCF7	1
2	1082	irinotecan	100 µM	MCF7	0.917
3	726	phenoxybenzamine	12 µM	MCF7	0.895
4	758	phenoxybenzamine	12 µM	MCF7	0.871
5	1091	irinotecan	100 µM	PC3	0.792
6	708	hycanthone	11 µM	MCF7	0.781
7	687	camptothecin	11 µM	MCF7	0.747
8	1084	daunorubicin	1 µM	MCF7	0.746
9	755	phenoxybenzamine	12 µM	MCF7	0.741
10	1088	daunorubicin	1 µM	MCF7	0.727

Topoisomerase I/II are main targets for antitumor drugs, and some studies have reported that NC inhibits topoisomerase activities^15–17^. However, few reports have conducted experiments on the simultaneous inhibition of topoisomerase I/II activities by NC. To validate the topoisomerase I/II inhibitory activity of NC, we evaluated the effect of NC on the stabilization of the cleavable complex that forms in the presence of topoisomerase I/II and DNA. As illustrated in Fig. 2b and c, NC was active against the topoisomerase I/II-mediated relaxation of supercoiled DNA. In addition, the effect of NC completely inhibited topoisomerase I at a concentration of 10 μM and completely inhibited the cleavage activity of topoisomerase II at 5 μM.

Furthermore, to validate the function of NC as an α-adrenoreceptor antagonist, we performed the classic adrenaline reversal experiment. α-Adrenoreceptor-blocking agents have the ability to cause a reversal of the adrenaline pressor response^18^. Adrenaline administration to animals results in an increased mean arterial blood pressure given that its α-receptor agonist properties predominate. However, when an α-adrenoreceptor antagonist is present, the β-receptor agonist property of adrenaline plays a leading role and causes a decrease in arterial pressure or reversal of the pressor response. As shown in Fig. 2d and e, after rats were injected with adrenaline (5 μg/kg), the arterial blood pressure increased abruptly. However, after pre-treatment with an injection of NC (0.1 mg/kg) followed by an injection of adrenaline (5 μg/kg), the arterial blood pressure significantly decreased. In addition, NC dose-dependently reduced the adrenaline pressor response (Fig. 2f). NC (0.1 mg/kg) caused a significant reversal of adrenaline pressor response with a reduction to normal levels (35 ± 1.4 mmHg).

In summary, most molecules have more than one effect, especially TCM molecules. The results of CMAP analysis showed that topoisomerase I/II inhibitor and α-adrenoreceptor antagonist produced high positive connectivity scores. After topoisomerase I/II inhibitory activity assays and adrenaline reversal experiments, NC was validated as an effective topoisomerase I/II inhibitor and candidate α-adrenoreceptor antagonist.

### Synergistic mechanism of TCM components

TCMs consist of numerous ingredients, and the therapeutic effect of TCMs mainly originates from the synergistic effect of these multiple components^19^. Synergy is one of the fundamental advantages of multicomponent therapeutics, indicating that combinational effects are greater than the sum of the individual effects^20, 21^. However, the mechanisms of synergistic action remain poorly understood. We attempted to conduct a systematic analysis to explore the rationality of the synergistic effects of the principal compounds in Danshen (*Salvia miltiorrhiza* roots). Danshen is one of the most versatile TCMs based on its properties of improving microcirculation, causing coronary vasodilatation, suppressing the formation of thromboxane, inhibiting platelet adhesion and aggregation, and protecting against myocardial ischemia, among other effects^22, 23^. Therefore, Danshen has been used to treat cardiovascular diseases, including coronary artery disease, hypercholesterolemia, hypertension, arrhythmias, and other cardiovascular diseases, for hundreds of years^23^.

In this work, we selected the four main active components (tanshinone IIA, salvianic acid A sodium, protocatechuic aldehyde and salvianolic acid B) in Danshen to elucidate their synergistic effect in the treatment of cardiovascular diseases. In this study, the differential expression levels of genes in MCF7 cells treated with each compound and four mixtures (Supplementary Data 2) were selected and analyzed in the CMAP database. Only drugs associated with cardiovascular diseases with positive connectivity scores and p < 0.01 were summarized (Table 3). The CMAP analysis results of four mixtures suggested that 11 drugs were related to cardiovascular diseases, including cardiovascular agents, cardiotonic agents, vasodilator agents, anti-arrhythmia agents, antihypertensive agents and calcium channel blockers. Most of these drugs ranked at or near the top, which indicated significant positive enrichment. However, the CMAP analysis results of single compounds indicated that fewer drugs were associated with cardiovascular diseases and received a lower ranking. The CMAP analysis results indicated that four mixtures possessed more positive effects on the therapeutic efficacy of cardiovascular diseases than any other individual components.

**Table 3:** Results of CMAP analysis.

	Rank	Cmap name	Enrichment	p	Therapeutic Use
Four mixtures	1	phenoxybenzamine	0.981	0	Antihypertensive Agents, Vasodilator Agents
	2	lanatoside C	0.981	0	Cardiovascular Agents
	3	digitoxigenin	0.978	0	Cardiotonic Agents
	5	digoxigenin	0.967	0	Cardiotonic Agents
	7	ouabain	0.962	0	Cardiotonic Agents
	8	digoxin	0.959	0	Anti-Arrhythmia Agents, Cardiotonic Agents
	28	proscillaridin	0.968	0.00004	Cardiotonic Agents
	60	beta-escin	0.663	0.00427	Cardiovascular Agents
	62	bepridil	0.778	0.00464	Anti-Arrhythmia Agents, Vasodilator Agents
	73	strophanthidin	0.738	0.00927	Cardiovascular Agents
	75	tetrandrine	0.733	0.00989	Calcium Channel Blockers
Tanshinone IIA	15	phenoxybenzamine	0.85	0.00072	Antihypertensive Agents, Vasodilator Agents
	17	lanatoside C	0.738	0.00085	Cardiovascular Agents
	23	beta-escin	0.711	0.00167	Cardiovascular Agents
	44	ouabain	0.733	0.00989	Cardiotonic Agents
Salvianic acid A sodium	16	timolol	0.761	0.00621	Anti-Arrhythmia Agents
	17	labetalol	0.761	0.00623	Antihypertensive Agents
	18	fenofibrate	0.847	0.00693	Hypolipidemic Agents
	20	propranolol	0.749	0.0076	Anti-Arrhythmia Agents, Antihypertensive Agents, Vasodilator Agents
protocatechuic aldehyde	12	pronetalol	0.771	0.00539	Antihypertensive Agents
	23	guanabenz	0.676	0.00927	Antihypertensive Agents
Salvianolic acid B	15	benfluorex	0.796	0.00338	Hypolipidemic Agents

We also applied the algorithm of random walk with restart (RWR) to elucidate the synergistic effects of multi-components of Danshen. RWR is a widely accepted algorithm that globally scores each gene in the entire network by computing the effects of seed genes^24–26^. Therefore, we computed the cardiovascular effect scores of the mixture of the four components and each single component separately. In addition, to verify whether their effect scores regarding cardiovascular disease were notable, we computed their Z-scores and compared them with random counterparts. The results are listed in Table 4. The mixture of the four components obtained a high effect score of 0.72, which was much higher than that of any single component. An absolute *Z*-score greater than 3 is generally deemed as a threshold, which suggests a statistically significant deviation between the actual value and the random values. Thus, the Z-score of 4.958 for the mixture of the four components was much higher than that of any single component, which suggested that the synergy among the four components is significantly associated with the effects of Danshen on cardiovascular disease. Therefore, the RWR algorithm showed that the synergistic effect of the mixture of the four components on multiple target genes outperforms the effects of single components.

**Table 4:** Effect scores and Z-scores of four mixtures and its single component to cardiovascular disease.

Components	Target number	Effect scores	Z-scores
Four mixtures	966	0.720	4.958
Tanshinone IIA	549	0.396	2.319
Salvianic acid A sodium	326	0.231	1.121
protocatechuic aldehyde	354	0.266	2.274
Salvianolic acid B	170	0.131	1.627

**Figure 3:**
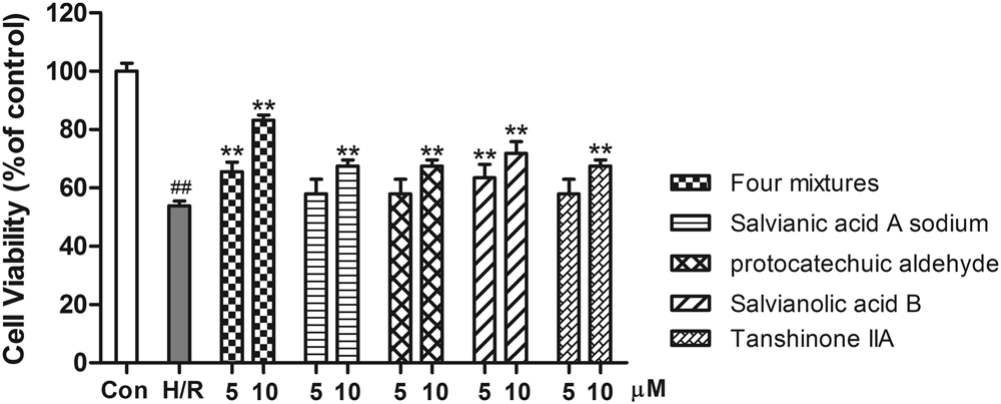
Effects of single compounds and four mixtures on H/R-induced H9c2 cell injury. Cell viability by CCK-8 assay. ^*##*^ *P* < 0.01 vs. Con; **P < 0.01 vs. H/R. Data are shown as the mean ± SD of three independent experiments.

In addition, cardioprotective effect assays were conducted on single compounds and four mixtures to validate the synergistic mechanisms of components. As showed in Fig. 3, hypoxia/reoxygenation (H/R)-induced cell injury significantly reduced cell viability. At concentration of 10 µM, all single compounds and four mixtures could notably protect H9c2 cells from H/R-induced cell injury. The cell viability of four mixtures was higher than that of any single compound. Thereby, four mixtures can exert more cardioprotective effect than single compounds, which sheds light on the synergistic therapeutic mechanisms of TCM components.

## Discussion

With the application of TCMs in various diseases receiving increasing attention worldwide, more and more efforts have been made to elucidate the mechanisms of TCMs. TCMs are highly diverse, with abundant ingredients, making TCMs a potential repository of molecules for drug discovery. Thus, developing an effective method to investigate the molecular mechanisms of TCMs is necessary. Since the emergence of microarray techniques, gene expression profiling has been widely used in the field of TCMs, and various achievements have been attained using this methodology. Lee *et al*.^27^ used gene expression profiles combined with the CMAP database to elucidate the mechanism of the Chinese herbal medicine berberine, which inhibits global protein synthesis and basal AKT activity and induces endoplasmic reticulum (ER) stress and autophagy. Li *et al*.^28^ adopted gene expression profiles to elucidate the multi-compound, multi-target and multi-pathway mechanism of action of a TCM formula, QiShenYiQi, on myocardial infarction. Therefore, gene expression profiles can provide new insight into the mechanisms of TCMs at the molecular and gene levels.

In this study, we established the gene expression profiles of cells in response to 102 TCM molecules, and this information will likely accelerate progress in understanding the molecular mechanisms of TCMs. Then, we used gene expression profiles combined with the CMAP database to explore the molecular functions of NC. After validation experiments, NC was identified as a topoisomerase I/II inhibitor and a potential new α-adrenoreceptor antagonist. This result indicates that some compounds in TCMs have more than one function and the approach is efficient. In addition, we analyzed the gene expression profiles of four active ingredients in Danshen and elucidated the theory of synergistic action in TCM multicomponent therapeutics. These results demonstrate the reliability and utility of our gene expression profile data. This approach provides an integrative platform to simultaneously analyze a large number of genes associated with TCM ingredients and offers convenience to researchers.

We only selected 102 molecules from TCMs, and these molecules only represent a small part of the numerous compounds in TCMs. In subsequent studies, we will further expand the number of small molecules. Such data will provide more possibilities for research on the molecular mechanisms of TCMs. For example, the theory of detoxification in TCMs is that it occurs mainly through herbal ingredient interactions, which can likely be elucidated by analyzing the gene expression profiles of molecules at the gene level. In the future, the gene expression profiles of cells in response to TCM components can help researchers to perform their own bioinformatics analyses to clarify the mechanisms of action of TCMs in real time. In short, this is just the beginning, and additional outcomes will depend on the use of the data.

## Methods

### The establishment of gene expression profiles of TCM components

#### The selection of components in TCMs

We selected 102 small molecules that are commonly found in Chinese herbs and TCM formulae, such as *Radix Salviae Miltiorrhizae*, *Rhizoma Coptidis*, and Shexiang Baoxin Pill. Most of these 102 compounds are the quality control components of TCMs from the China Pharmacopoeia and are selected to represent a broad range of activities and diverse structures.

#### Cell lines

The gene expression profile data for 102 molecules were produced for MCF7 cells. MCF7 cells are commonly used in the worldwide laboratories as a reference cell line, have clear biological characteristics, remain stable after prolonged culture, and can be cultured in microplates. In addition, MCF7 is the one of the main cell lines used in the CMAP database^4^. Furthermore, the MCF7 cell line was procured from American Type Culture Collection (ATCC) and cultured in MEM/EBSS (Hyclone) supplemented with 10% foetal bovine serum, 1 mmol/L sodium pyruvate, 0.1 mmol/L MEM non-essential amino acids, 100 unit/mL penicillin, and 100 mg/mL streptomycin in an incubator containing 5% CO_2_ at 37 °C.

#### Small molecule-treated cells

The gene expression profiles can be affected by the concentration and duration of compound treatment. According to the CMAP database, the concentration of small molecules was set at a single dose of 10 μM, which is also internationally recognized as a reasonable concentration for high-throughput screening^4^. Simultaneously, the cell’s survival rate was investigated upon treatment with compounds at a concentration of 10 μM using the MTT assay. If the cell survival rate was <40%, the number of cells could not meet the needs of microarray analysis. Therefore, the concentration of compounds was decreased to 1 μM until the cell survival rate was >40%. For each compound, the duration of the treatment was 12 h and two biological replicates were performed. Full details of small molecules and treatment conditions are provided in Table 1.

#### RNA isolation and quality

After pre-treatment, MCF7 cells were harvested and total RNA was extracted using TRIzol reagent (Life Technologies, Carlsbad, CA, US) according to the manufacturer’s instructions. To control the quality and purity of isolated total RNA, formaldehyde agarose gel electrophoresis and spectrophotometry (NanoDrop, Wilmington, DE, USA) were performed. Moreover, DMSO-treated cells were selected as a control.

#### Microarray analysis

The gene expression profiles were assessed using microarray technology with Affymetrix Human Genome U133A 2.0 (Santa Clara, CA, US), which was used in numerous studies, covering 18,400 transcripts and including 14,500 characterized human genes^29–31^. Total RNA was purified using a QIAGEN RNeasy Kit (GmBH, Germany) according to the manufacturer’s protocols, and biological duplicates were employed for each cell line. Then, total RNA was used to generate double-stranded cDNA and biotin-labelled cRNA. Following fragmentation, cRNA products were hybridized to an Affymetrix Human Genome U133A 2.0. GeneChip array, and hybridized arrays were washed and stained using a GeneChip® Hybridization, Wash and Stain Kit (Affymetrix). Finally, the fluorescent signals were measured with the GeneChip® Scanner 3000 (Affymetrix).

The data from this publication have been deposited in NCBI’s Gene Expression Omnibus (GEO series accession number: GSE85871). Raw data (CEL files) were normalized by MAS 5.0 algorithm, Gene Spring Software 11.0 (Agilent Technologies, Santa Clara, CA, US). Subsequently, quality control (QC) analysis was performed on the expression data, including overview of QC analysis, quality on-chip analysis, comparative analysis among multiple samples, PCA, and RNA degradation analysis, by using the affy package in R language. The results indicated that all data met the requirements for bioinformatics analysis.

### Similarity search against the CMAP

In the investigation of the function of a small molecule, a similarity search against the CMAP was performed. For each treatment of one compound (one treated versus the corresponding DMSO pair), Fold Change was used to filter the differential expression probes which was calculated as follows: first of all, average normalized expression values were calculated for two replications for each small molecule and DMSO; second, Fold Change was represented by average normalized expression value of treatment divided by the average normalized expression value corresponding to DMSO. Then, differential expression probes were selected according to fold change (e.g., FC ≥2 or ≤0.5), the criteria used for filtering the differential expression probes were consistent among the small molecules. The gene-expression signature of the compound was represented by two sets (‘up-’ and ‘down-’ probe sets, saved as. grp files and required as the inputs for CMAP), which was made up by the significant up/down regulation probes respectively. The query in the CMAP was performed as a “quick query” in the query section of http://portals.broadinstitute.org/cmap/.

### Topoisomerase I/II inhibitory activity assay

Topoisomerase I/II inhibitory activity assays were conducted according to the procedure described in a previous study^32^. Compound concentrations, pBR322 plasmid DNA (0.25 μg) and 1 unit of TopI (TaKaRa Biotechnology Co., Ltd., Dalian) were combined in a final volume of 20 μL buffer (35 mM pH 8.0 Tris-HCl, 72 mM KCl, 5 mM MgCl_2_, 5 mM dithiothreitol, 5 mM spermidine, 0.1% bovine serum albumin). Then, the mixed reaction buffers were incubated for 15 min at 37 °C and stopped by the addition of 2 μL of 10× loading buffer. The samples were analyzed by electrophoresis on a 0.8% agarose gel in TAE (Tris-acetate-EDTA) for 1 h and then stained with 0.5 μg/mL of ethidium bromide for 30 min. Finally, the DNA band was visualized using UV light and photographed with a *G*:*BOX* gel imaging system (Gene Co., Ltd., Hong Kong).

The DNA TopIIα inhibitory activity of the compounds was measured using a Topoisomerase IIα Drug Screening Kit (TopoGEN, Inc.). Compound concentrations, pBR322 plasmid DNA (0.25 μg) and 0.75 unit of TopII were combined in a final volume of 20 μL buffer (50 mM pH 8.0 Tris-HCl, 150 mM NaCl, 10 mM MgCl_2_, 5 mM dithiothreitol, 30 μg/Ml bovine serum albumin, 2 mM ATP). Then, the following experimental procedures were performed according to Topoisomerase I inhibitory activity assay.

### Adrenaline reversal experiments

Male Sprague-Dawley rats (300–350 g) were obtained from the Slac Laboratory Animal Co., Ltd. (Shanghai, China), and given free access to tap water and food pellets. All animal experiments were carried out under standard conditions according to the guidelines for the Care and Use of Laboratory Animals of the National Institutes of Health and were approved by the Committee on the Ethics of Animal Experiments of the Second Military Medical University, China. The animals were anesthetized with urethane (1 g/kg) with minimal suffering. Mean blood pressure was continuously monitored from a cannulated carotid artery using a pressure transducer to a polygraph (Alcott Biotech Co., Ltd. Shanghai). All care was taken that animals could breathe normally. Adrenaline and NC were administered through a catheter inserted into the tail vein. Experiments were performed only after completion of the operative procedures to permit arterial blood pressure to stabilize.

Animals were randomly divided into five groups. Group A rats received a single injection of adrenaline (5 μg/kg). Group B, C, and D rats received an injection of NC (0.01, 0.025, and 0.1 mg/kg, respectively) after a 2-min injection of adrenaline (5 μg/kg). Group E rats received a single injection of NC (0.1 mg/kg). After the experiments, all animals were sacrificed by tail vein air injection.

### RWR-based evaluation of compounds’ effect

#### Data preparation

In total, 301 distinct genes associated with cardiovascular diseases were collected by searching the key word “Cardiovascular Disease” in a plugin of Cytoscope, DisGeNet. In addition, Version 10 of the STRING database was employed as a resource of the PPI network. We extracted interactions with confidence scores greater than 0.9 or target-related edges with maximum scores. Thus, a PPI network was constructed, and its greatest weighted component had 10,270 nodes and 176,739 edges.

#### RWR algorithm

RWR can globally score seed genes’ effects on each gene in the entire network and can be denoted as follows:

1 $${{\chi }}^{{t}+{1}}=({\rm{1}}-{r}){P}{{\chi }}^{{t}}+{r}{{\chi }}^{{0}},$$

where P is the column-normalized adjacency matrix of the network, *χ* ^0^ is the initial vector that indicates the seed nodes’ strength, and *χ* ^*t*^ denotes a probability vector in which the *i*th element holds the chance of the walker being at *v*_*i*_ node at step t. Parameter r indicates a restart probability indicating the likelihood that the walker will return to seed set at step t. In practice, 0.3 is an optimal value. A steady state of *χ* ^*t*^ will be reached after performing the eq. (1) iteratively with sufficient time, which can disclose to which extent each node is affected by seed nodes.

In this paper, disease genes and differentially expressed genes under the treatment of the molecules are regarded as seed nodes separately. To calculate disease effect, the corresponding component of the disease gene in the initial vector is *χ* ^*0*^(*v*) = 1. To compute drug effect, if node *v* is a drug target, we define its corresponding component in the initial vector *χ* ^*0*^ as *χ* ^*0*^(*v*) = 0.01. After running the RWR with these initial vectors, we obtain the drug and disease effect vectors *χ*_*drug*_ and *χ*_*disease*_, respectively. Then, we compute the inner product between the effect vectors of drug and disease to measure how the drug-affected network and disease-affected network overlap. The equation is

2 $${s}=\langle {{\chi }}_{{disease}}{,}{{\chi }}_{{drug}}\rangle .$$

To measure the statistical significance of the score *s*, we randomly generate 1000 counterparts that have the same number of drug targets and calculate *s* scores. Suppose $\bar{{s}}$ and Δ*s*_*r*_ are the mean and standard deviation of these random counterparts’ scores, respectively. Then, the z-score can quantify the score difference among the original seed set and counterparts as follows:

3 $${\rm{Z}}=\frac{{s}-{\bar{{s}}}_{{r}}}{{\rm{\Delta }}{{s}}_{{r}}}.$$

Typically if the z-score is greater than 3, the drug can be considered as exhibiting statistically stronger effects than random cases.

### Cardioprotective effect assay

Cardioprotective effect assays were performed by determing the effects of single compounds and four mixtures against H/R-induced H9c2 cells injury. Rat H9c2 cardiomyocyte cell line was obtained from Chinese Academy of Sciences Cell Bank (Shanghai, China) and maintained in DMEM supplemented with 10% foetal bovine serum at 37 °C in CO_2_ incubation. To mimic the ischemic injury *in vitro*, H9c2 cells were placed in a humidifed chamber containing the cells with 95% N_2_ and 5% CO_2_ for 4 h and maintained in serum-free and glucose-free DMEM. Then, the cells were transferred to normal conditions for 20 h and cultured in routine culture medium to achieve reoxygenation. The single compounds and four mixtures (1:1:1:1) were added 1 h before the hypoxia period. Cell viability was determined by Cell Counting Kit-8 assay (CCK-8; Dojindo, Kumamoto, Japan).

## Electronic supplementary material

Dataset 1

Dataset 2
